# Does Molecularly Guided Therapy Change the Surgical Goals of Glioma Resection?

**DOI:** 10.1002/brb3.71913

**Published:** 2026-11-06

**Authors:** Naeem Ul Haq, Sardar Umer Irshad, Irfan Ullah, Sardar Remsha Irshad, Shah Mir, Muhammad Shaheer Khan, Ali Mumtaz, Malik Ahsan Jamal, Jahan Mubariz

**Affiliations:** ^1^ Department of Neurosurgery Bacha Khan Medical College Mardan Pakistan; ^2^ Department of Medicine Khyber Girls Medical College Peshawar Pakistan; ^3^ Khyber Medical College Peshawar Pakistan; ^4^ Kabul University Kabul Afghanistan

**Keywords:** CAR‐T cell therapy, extent of resection, glioma surgery, IDH‐mutant glioma, surgical decision‐making

## Abstract

**Purpose:**

For more than 20 years, the goal of glioma surgery has been to remove as much tumor as possible without compromising patient safety. Many retrospective studies support that greater extent of resection is often associated with longer survival. However, three recent developments have challenged this traditional approach. First, the 2021 World Health Organization classification introduced a molecular approach to glioma diagnosis, which made tumor biology an additional consideration, alongside imaging, in setting surgical targets. Second, the IDH‐targeted therapies, particularly vorasidenib for IDH‐mutant low‐grade gliomas, have made residual disease a clinically meaningful variable rather than a surgical failure. Third, early phase locoregional chimeric antigen receptor (CAR) T‐cell trials have suggested that the resection cavity may be an important site for treatment delivery. These developments raise a question: should the goal of glioma surgery remain the same when targeted and locoregional therapies are available? In this review, we examine the evidence for extent of resection and discuss how newer therapies may affect surgical decision‐making.

**Methods:**

A structured literature search of PubMed, Scopus, and Web of Science (inception to May 2026) was conducted, and evidence quality was graded narratively.

**Findings:**

We argue that these therapies do not replace maximal safe resection but reshape its application in clinical practice. Surgical planning may increasingly need to consider tissue sampling and access for future therapies alongside tumor removal. We also highlight five key controversies and outline five priorities for future research.

**Conclusion:**

Based on the available evidence, we propose an oncofunctional‐immunotherapeutic framework in which molecular subtype is considered before surgery, the resection cavity is seen as a potential therapeutic site, and preservation of neurological function remains essential for enabling further treatment. These elements are not yet integrated into a single clinical framework.

**Abbreviations**
5‐ALA: 5‐aminolevulinic acid
ATRX: alpha thalassemia/mental retardation syndrome X‐linked
BBB: blood‐brain barrier
CAR‐T: chimeric antigen receptor T‐cell
CDKN2A/B: cyclin‐dependent kinase inhibitor 2A/B
CE: contrast‐enhancing
CNS: central nervous system
CSF: cerebrospinal fluid
DES: direct electrostimulation
EGFR: epidermal growth factor receptor
EGFRvIII: EGFR variant III
EOR: extent of resection
FISH: fluorescence in situ hybridization
GBM: glioblastoma multiforme
GTR: gross total resection
ICANS: immune effector cell‐associated neurotoxicity syndrome
IDH: isocitrate dehydrogenase
IL‐13Rα2: interleukin‐13 receptor alpha 2
LGG: low‐grade glioma
MDSC: myeloid‐derived suppressor cell
MEP: motor evoked potential
MGMT: O6‐methylguanine‐DNA methyltransferase
MRI: magnetic resonance imaging
nCE: non‐contrast‐enhancing
NGS: next‐generation sequencing
NSC: neural stem cell
OS: overall survival
PFS: progression‐free survival
STR: subtotal resection
TAM: tumor‐associated macrophage
TIAN: tumor inflammation‐associated neurotoxicity
TIL: tumor‐infiltrating lymphocyte
TME: tumor microenvironment
TMZ: temozolomide
Treg: regulatory T cell
WHO: World Health Organization

## 1 Introduction

Gliomas account for approximately 80% of malignant brain tumors and represent a biologically heterogeneous group of diseases (Pöhlmann et al. 2024). Glioblastoma accounts for nearly 58% of glioma cases and carries poor survival outcomes (Zhao et al. 2025). Grade 2 IDH‐mutant gliomas, the population in which vorasidenib has been tested, often follow a slower but progressive course (Mellinghoff et al. 2023), although IDH‐mutant gliomas span CNS WHO grades 2 to 4, and grade 4 astrocytomas behave more aggressively.^4^ Health‐related quality of life remains a major treatment priority for many patients, making functional preservation an important goal of surgery (Pöhlmann et al. 2024).

For decades, glioma surgery was guided mainly by anatomical and histological findings. Surgery was consistently associated with improved survival, and gross total resection showed better outcomes than limited resection in large retrospective analyses (Zhao et al. 2025). Operative success was largely measured by postoperative residual tumor volume on MRI.

The 2021 WHO classification reorganized adult diffuse gliomas into molecularly defined entities and established integrated molecular diagnosis as standard practice (Louis et al. 2021). Under this system, IDH‐mutant astrocytoma is typically defined by ATRX loss and TP53 mutation, oligodendroglioma by 1p/19q codeletion with TERT promoter mutation, and IDH‐wildtype glioblastoma by features such as TERT promoter mutation, EGFR amplification, or +7/−10.^4^ H3 G34‐mutant tumors are classified as pediatric‐type diffuse hemispheric gliomas and fall outside this adult‐focused review.^4^ IDH mutation, MGMT promoter methylation, and CDKN2A/B homozygous deletion now influence prognosis and treatment planning (Louis et al. 2021; Juratli et al. 2023). Temozolomide‐based chemoradiotherapy remains the therapeutic backbone for glioblastoma management (Stupp et al. 2005). The INDIGO trial later demonstrated that vorasidenib prolonged progression‐free survival in grade 2 IDH‐mutant glioma and introduced new considerations regarding timing of surgery and management of residual disease (Mellinghoff et al. 2023). Early‐phase trials of locoregional chimeric antigen receptor T‐cell therapy have also increased the importance of operative access, catheter placement, and tissue acquisition in glioblastoma management (Brown et al. 2024; Geurts and Preusser 2024; Choi et al. 2024).

This narrative review examines how three developments, molecular classification, IDH‐directed therapy and locoregional immunotherapy, have unsettled long‐standing surgical assumptions in glioma and where they pull operative decisions in opposing directions: between maximal cytoreduction and the preservation of measurable disease for drug monitoring and between surgery as tumor removal and surgery as a delivery platform for immunotherapy. This raises a central question: should the goals of glioma surgery remain unchanged when targeted and locoregional therapies become available? Synthesizing this evidence, we propose an oncofunctional‐immunotherapeutic framework in which molecular subtype is treated as a preoperative planning variable, the resection cavity as a therapeutic instrument, and functional preservation as a prerequisite for downstream therapy rather than a secondary goal.

## 2 Methodology

### 2.1 Review Design and Reporting Framework

This article is a narrative review with a structured and transparent literature search. It aims to critically synthesize evidence on how molecular classification, IDH‐directed therapy, and locoregional immunotherapy influence surgical decision‐making in glioma. A systematic review or meta‐analysis was not undertaken because the available evidence is heterogeneous in design, population, and outcome measures, and the aim was conceptual synthesis rather than pooled effect estimation. As a narrative review, it was not prospectively registered, since registration platforms such as PROSPERO do not accept narrative reviews.

### 2.2 Literature Search

PubMed/MEDLINE, Scopus, and Web of Science were searched from database inception to May 2026. No lower date limit was applied so that landmark extent‐of‐resection studies could be captured. ClinicalTrials.gov was searched to identify early‐phase locoregional CAR‐T trials. Search terms combined controlled vocabulary and free text across four domains using Boolean operators:

- Disease: glioma, glioblastoma, IDH‐mutant glioma, and low‐grade glioma
- Surgery: extent of resection, gross total resection, supratotal resection, maximal safe resection, intraoperative MRI, 5‐aminolevulinic acid, and awake craniotomy
- Molecular and targeted therapy: WHO 2021 classification, IDH mutation, vorasidenib, MGMT, and CDKN2A/B
- Immunotherapy: CAR‐T, locoregional delivery, intraventricular, resection cavity, and tumor microenvironment

An example PubMed string was *(glioma OR glioblastoma) AND (“extent of resection” OR “supratotal resection” OR “maximal safe resection”) AND (IDH OR vorasidenib OR “CAR‐T” OR “resection cavity”)*. Reference lists of key articles and relevant guidelines (WHO 2021, EANO, NCCN, SNO) were hand‐searched to identify additional sources.

### 2.3 Eligibility Criteria

Eligible sources were English‐language, peer‐reviewed publications on adult diffuse glioma, including randomized trials, prospective and retrospective cohort studies, phase 1–3 clinical trials, systematic reviews, meta‐analyses, narrative and consensus reviews, clinical practice guidelines, and classification documents. Selected preclinical or translational studies were also included where they informed the mechanistic rationale for locoregional therapy. Topics covered were extent of resection, molecular classification, targeted therapy, locoregional immunotherapy, intraoperative technologies, functional outcomes, and disease burden. Preference was given to the highest‐level and most recent evidence. Case reports, conference abstracts, preprints, non‐peer‐reviewed gray literature as sources of evidence, non‐English publications, and studies limited to pediatric gliomas were excluded. Editorials and commentaries were not used as primary evidence and were cited only to provide expert interpretation alongside the primary studies they discuss. Sources on non‐glioma tumors or general neurosurgical topics were included only where they directly informed a glioma‐specific surgical question.

### 2.4 Study Selection and Data Extraction

Titles and abstracts were screened for relevance by the lead author, followed by full‐text assessment of potentially eligible articles. Uncertain cases were discussed with the senior author before a final decision was made. For each included source, the following were extracted: study design, sample size, glioma grade and molecular subtype, extent‐of‐resection target or intervention, key outcome, and main limitation. Key studies are summarized in Tables 1, 3, and 5. Because this is a narrative review, screening was not performed in duplicate, no PRISMA flow diagram was produced and no quantitative pooling was performed.

**TABLE 1:** Extent of resection in glioma: landmark studies and evidence credibility.

Author (Year) [Ref]	N	Design	Grade	EOR target	Key outcome	Evidence class / credibility	Primary limitation
Lacroix et al. (2001) (Lacroix et al. 2001)	416	Retrospective	GBM	≥98% EOR	OS 13.0 vs. 8.8 mo (p < 0.0001)	Class III; low	No molecular data; statistical bias
Sanai et al. (2011) (Sanai et al. 2011)	500	Retrospective, volumetric	GBM	≥78% EOR	Stepwise OS benefit up to 100% EOR (p < 0.0001)	Class III; low	Selection bias
Sanai and Berger (2008) (Sanai and Berger 2008)	38 studies	Systematic review	HGG, LGG	Greater EOR	Greater resection linked to longer survival	Class III; low	Narrative synthesis
Brown et al. (2016) (Brown et al. 2016)	41,117	Meta‐analysis	HGG	GTR vs. STR	1‐yr mortality RR 0.62 (95% CI 0.56–0.69); NNT = 9	Class III; low	I^2^ = 91.9%; excess significance bias
Jagtiani et al. (2024) (Jagtiani et al. 2024)	43 studies	Umbrella review	HGG	GTR vs. STR	eG = −0.356 (95% CI −0.554 to −0.158; p = 0.0004)	Class III; low	High heterogeneity
Xia et al. (2018) (Xia et al. 2018)	2,128	Meta‐analysis	LGG	GTR	OR 3.90 (5‐yr OS); OR 10.17 (10‐yr OS vs. biopsy)	Class III; moderate	Retrospective primaries; inconsistent T2‐FLAIR
Duffau (2013) (Duffau 2013)	15	Prospective case series	LGG	Supra‐total	Zero anaplastic transformation vs. 7 in GTR control (mean FU 35.7 mo)	Class IV; low	Small sample
Molinaro et al. (2020) (Molinaro et al. 2020)	761	Retrospective	IDH‐wt GBM	Maximal CE + nCE	Median OS 37.3 month	Class III; low	Retrospective design
De Witt Hamer et al. (2012) (De Witt Hamer et al. 2012)	>8,000	Meta‐analysis	HGG, LGG	Awake mapping + DES	Reduced permanent deficit with increased EOR	Class III; moderate	Heterogeneous techniques
Stummer et al. (2006) (Stummer et al. 2006)	322	Phase III RCT	GBM	5‐ALA guided	Complete resection 65% vs 36%; 6‐mo PFS 41% vs. 21% (p = 0.0003)	Class I; high	Radiographic definition only

Evidence class and certainty were assigned by the authors using the pragmatic scheme in Section 2.5.

Abbreviations: 5‐ALA, 5‐aminolevulinic acid; CE, contrast‐enhancing; CI, confidence interval; DES, direct electrostimulation; EOR, extent of resection; FU, follow‐up; GBM, glioblastoma; GTR, gross total resection; HGG, high‐grade glioma; IDH‐wt, IDH‐wildtype; LGG, low‐grade glioma; mo, months; nCE, non‐contrast‐enhancing; NNT, number needed to treat; OR, odds ratio; OS, overall survival; PFS, progression‐free survival; RCT, randomized controlled trial; RR, relative risk; STR, subtotal resection.

### 2.5 Evidence Appraisal

Formal risk‐of‐bias tools were not applied, as is usual for narrative reviews. Instead, each key source was assigned a pragmatic evidence class according to design (Class I, randomized trial; Class II, prospective cohort study; Class III, retrospective study or meta‐analysis of observational data; Class IV, small uncontrolled case series) and a certainty rating (high, moderate, or low), informed by GRADE principles. This considered design, sample size, selection bias, heterogeneity, imprecision, and applicability to current molecularly defined glioma. These ratings are shown in the “Evidence class/credibility” and “Primary limitation” columns of Table 1. Conclusions drawn from retrospective or early‐phase data are worded as hypotheses (“may”, “suggests”) rather than established findings. Selection and appraisal were performed by a single reviewer, with uncertain cases discussed with the senior author, and without independent duplicate verification. This is acknowledged as a limitation (Section 8.4).

### 2.6 Synthesis

Evidence was synthesized thematically under the review's core domains: extent of resection, molecular stratification, targeted therapy, immunotherapy, and functional preservation. Areas of disagreement were identified as controversies (Section 6), and the proposed oncofunctional‐immunotherapeutic framework was developed from this synthesis. The framework is a conceptual proposal by the authors and has not been prospectively validated.

## 3 The Resection Paradigm

### 3.1 The Foundational Case for Maximal Resection

The survival benefit of the extent of resection in glioma is supported mainly by retrospective evidence. Lacroix et al. reported longer survival in patients with at least 98% tumor resection in a 416‐patient glioblastoma study (2001). Sanai et al. later reported a progressive survival association beginning at 78% extent of resection, which may suggest a dose‐response relationship rather than a strict cutoff (2011). Supporting this, Sanai and Berger's review of 38 glioma studies associated greater resection with prolonged survival in both high‐ and low‐grade gliomas, despite the absence of Class I evidence (Sanai and Berger 2008).

Meta‐analyses have reported improved survival with gross total resection in both high‐grade and low‐grade glioma, although evidence credibility remains low to moderate as summarized in Table 1 (Brown et al. 2016; Jagtiani et al. 2024; Xia et al. 2018). Duffau further proposed that MRI underestimates LGG infiltration, supporting the rationale for supra‐total resection beyond radiographic margins (2013). In low‐grade glioma, supra‐total resection beyond MRI‐defined abnormalities was also associated with delayed malignant transformation in small prospective series (Yordanova et al. 2011).

These survival associations must be interpreted with caution. Almost all of this evidence is retrospective, and no randomized trial has tested the extent of resection directly, which leaves the literature open to several systematic biases. Patients selected for more aggressive resection tend to be younger, with better performance status and more favorably located tumors, so part of the apparent benefit reflects confounding by indication rather than the effect of resection itself. Survival measured from the date of surgery is also susceptible to immortal‐time bias, and heterogeneity across series is substantial, as reflected in the wide confidence intervals and high *I* ^2^ values summarized in Table 1. In these studies, the extent of resection was estimated volumetrically from contrast‐enhancing tumor on postoperative imaging, which does not capture non‐enhancing infiltration. Later work incorporated non‐enhancing tumors,^1^ ^8^ and the RANO resect classification (Section 8.2) is intended to standardize assessment of extent of resection in future studies. The consistency of the association across many cohorts is reassuring, but it does not establish causation, and the survival benefit attributable to resection alone remains uncertain.

### 3.2 Molecular Stratification of EOR Targets

The molecular era has changed traditional concepts of the extent of resection. Molinaro et al. reported that maximal resection of both contrast‐enhancing and non‐enhancing tumors was associated with longer survival in younger IDH‐wildtype glioblastoma patients but not in patients older than 65 years (2020). These findings suggest that operative targets should be interpreted in the context of tumor biology and patient factors.

Most foundational extent of resection studies were conducted before routine molecular profiling and did not account for biomarkers such as IDH mutation, MGMT methylation, or ATRX status (Hervey‐Jumper and Berger 2016). With molecularly targeted therapies now available, operative planning increasingly depends on obtaining adequate tissue for molecular characterization and treatment selection. The practical consequence is that the resection targets validated in the pre‐molecular era cannot be assumed to transfer unchanged to molecularly defined subgroups, where the relationship between extent of resection and outcome is only beginning to be characterized.

### 3.3 Functional Preservation and Intraoperative Adjuncts

Maximal safe resection requires balancing tumor removal with neurological preservation (Roh and Kim 2023). Awake craniotomy with direct electrostimulation is widely regarded as the preferred approach for eloquent cortex surgery and has been associated with improved extent of resection and lower permanent neurological deficits (Duffau 2013; Hervey‐Jumper and Berger 2016; De Witt Hamer et al. 2012). Fluorescence‐guided surgery with 5‐aminolevulinic acid was associated with higher complete resection rates and improved 6‐month progression‐free survival in a phase III trial, without a demonstrated overall survival benefit and without increasing neurological morbidity (Stummer et al. 2006).

Current evidence supports an individualized approach in which the extent of resection, operative technique, and preservation strategies are adapted according to tumor location, imaging features, and molecular subtype. While maximal safe resection remains the operative foundation of glioma surgery, contemporary planning increasingly requires integration of molecular biomarkers, targeted therapies, and treatment sequencing considerations. These principles are summarized in Figure 1. This shifts the question from whether to resect maximally to what “maximal” should mean once tumor biology, rather than imaging alone, defines the target.

**FIGURE 1:**
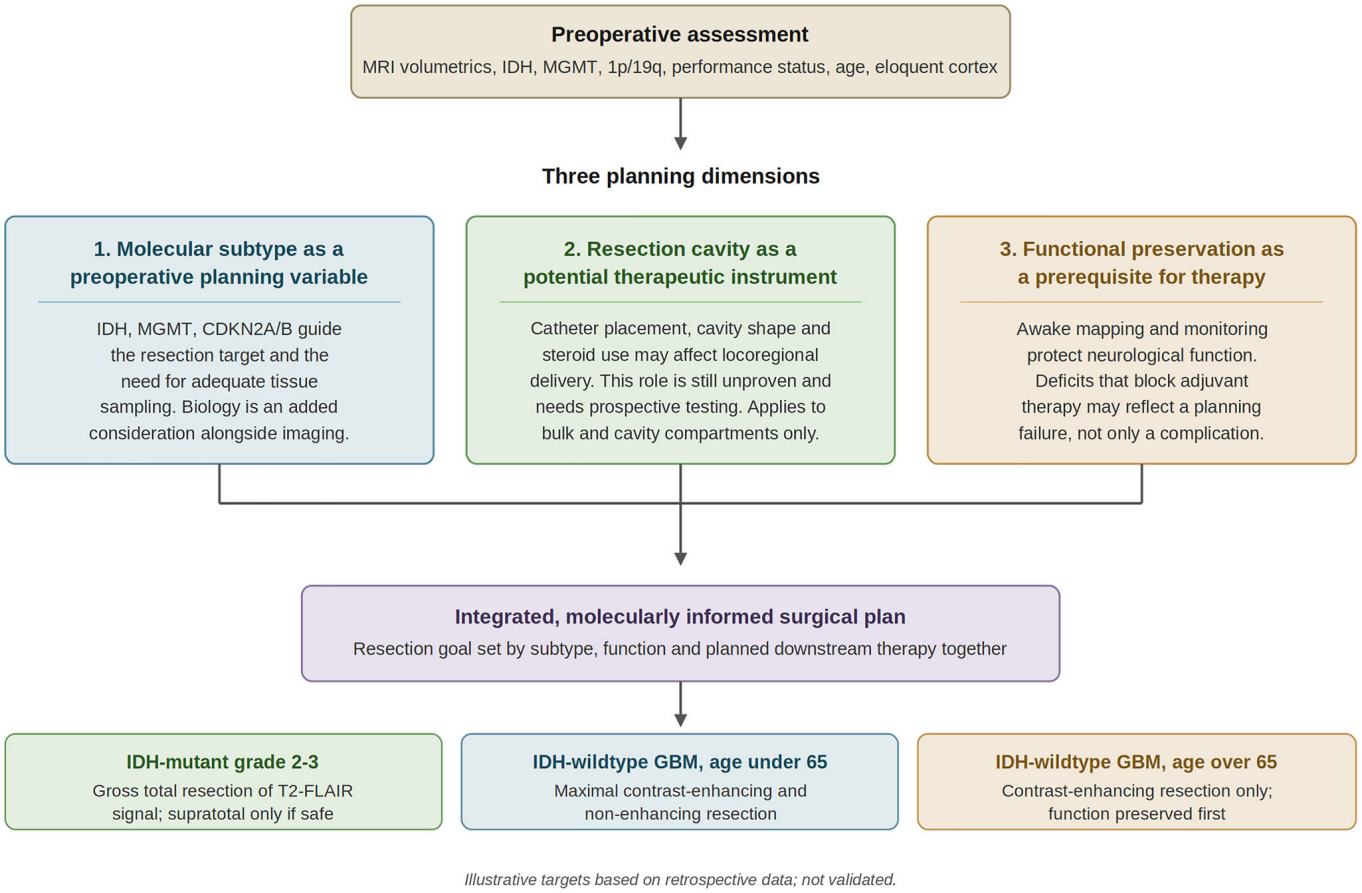
Surgical Decision‐Making Framework in Glioma: A Conceptual Overview. Alt text: Flowchart of a maximal safe resection framework in glioma surgery. Preoperative assessment (MRI volumetrics, IDH, MGMT, 1p/19q, performance status, age, and eloquent cortex) feeds three planning dimensions: molecular subtype as a preoperative variable (IDH, MGMT, and CDKN2A/B define the resection target by biology, not imaging); resection cavity as a potential therapeutic instrument (catheter placement, cavity shape, and steroid use may affect locoregional delivery, though unproven and requiring prospective testing); and functional preservation as a prerequisite for therapy (awake mapping protects neurological function; deficits blocking adjuvant therapy represent a planning failure). These converge into an integrated, molecularly informed surgical plan, stratified into three targets: IDH‐mutant grade 2–3 (gross total resection of T2‐FLAIR, supratotal only if safe), IDH‐wildtype GBM under 65 (maximal contrast‐enhancing and non‐enhancing resection), and IDH‐wildtype GBM over 65 (contrast‐enhancing resection only, function preserved first). Labeled a conceptual, non‐validated synthesis.

## 4 The Molecular Revolution

### 4.1 WHO 2021 Classification and Surgical Consequences

The 2021 WHO Classification reorganized adult diffuse gliomas into molecularly defined entities, including astrocytoma IDH‐mutant, oligodendroglioma IDH‐mutant, 1p/19q‐codeleted, and glioblastoma IDH‐wildtype (Louis et al. 2021). Molecular biomarkers now directly influence diagnosis, grading, and prognosis.

These changes have important surgical implications. Adequate tissue sampling is now essential for molecular analysis and treatment planning (Juratli et al. 2023; Guo et al. 2024). Imaging signs such as the T2‐FLAIR mismatch sign may support diagnosis but remain insufficiently sensitive for reliable classification without molecular confirmation (Park et al. 2023).

Table 2 summarizes the molecular classification system and its surgical implications.

**TABLE 2:** WHO 2021 molecular classification of adult diffuse gliomas and surgical consequences.

Tumor type	Defining molecular biomarkers	WHO grade	Key prognostic implication	Surgical consequence
Astrocytoma, IDH‐mutant	IDH1/2 mutation; ATRX loss; no 1p/19q codeletion	2, 3, 4	Favorable vs. IDH‐wt; CDKN2A/B deletion upstages to Grade 4	Tissue must yield a diagnostically complete molecular specimen for grading
Oligodendroglioma, IDH‐mutant and 1p/19q‐codeleted	IDH1/2 mutation + 1p/19q codeletion; TERTp mutation	2, 3	Best prognosis among diffuse gliomas; chemo‐sensitive	GTR of T2‐FLAIR target; 1p/19q status required for treatment eligibility often pursued where safe (retrospective evidence)
Glioblastoma, IDH‐wildtype	IDH‐wt plus ≥1 of: TERTp mutation, EGFR amplification, +7/−10	4	Median OS ∼15 months; MGMT methylation predicts TMZ response	Retrospective data suggest benefit of CE + nCE resection in younger patients but not > 65 years
IDH‐wt astrocytoma (molecularly upgraded)	Histologically low‐grade but TERTp+ / EGFR amp / +7−10	4 (GBM)	Morphologically deceptive; aggressive biology despite low‐grade look	Resection extent should not rely on imaging alone; molecular profiling mandatory
IDH‐mutant Grade 4 astrocytoma	IDH mutation + CDKN2A/B homozygous deletion	4	Worse than Gr 2/3 IDH‐mutant; better than IDH‐wt GBM	Maximal safe resection generally favored; supporting data are largely retrospective

Abbreviations: CE, contrast‐enhancing; EGFR, epidermal growth factor receptor; FISH, fluorescence in situ hybridization; GBM, glioblastoma; GTR, gross total resection; IDH, isocitrate dehydrogenase; IDH‐wt, IDH‐wildtype; MGMT, O6‐methylguanine‐DNA methyltransferase; nCE, non‐contrast‐enhancing; NGS, next‐generation sequencing; OS, overall survival; TERTp, TERT promoter; TMZ, temozolomide.

### 4.2 Targeted Therapy and Strategic Tension With Cytoreduction

The INDIGO trial demonstrated that vorasidenib significantly prolonged progression‐free survival in grade 2 IDH‐mutant glioma (Mellinghoff et al. 2023). At the same time, residual postoperative tumor volume remains an important prognostic factor in IDH‐mutant disease (Van Den Bent et al. 2024). This creates a clinical question regarding the balance between aggressive cytoreduction and preservation of measurable disease for targeted therapy monitoring.

Additional prognostic biomarkers, including CDKN2A/B deletion, PDGFRA amplification, and PI3K mutations, also require adequate tissue sampling for assessment (Tesileanu et al. 2021). Surgery therefore remains essential for molecular characterization and treatment planning.

Synthesizing available evidence, a pragmatic position emerges: in patients with grade 2 IDH‐mutant glioma where gross total resection is achievable without functional compromise, maximal cytoreduction may remain appropriate, given the prognostic value of residual tumor volume. Where gross total resection is not safely achievable, residual disease provides both a measurable target for vorasidenib monitoring and a rationale for early drug initiation per the ASCO‐SNO rapid update (Blakeley et al. 2025). The INDIGO trial enrolled patients with residual or recurrent disease; benefit from vorasidenib in molecularly complete resection remains unstudied and should not be assumed (Mellinghoff et al. 2023; Blakeley et al. 2025). Van den Bent et al. further reported that residual tumor volume retained independent prognostic significance in IDH‐mutant disease even in the targeted therapy era, which supports the oncological rationale for maximal cytoreduction where functionally feasible (2024).

The intermediate‐risk candidate profile for vorasidenib eligibility includes preserved performance status, non‐enhancing disease, and low preoperative tumor volume (Dipasquale et al. 2024). Preliminary pilot data from a small early‐access series in WHO grade 3 IDH‐mutant glioma reported 6‐month progression‐free survival of 90% with vorasidenib under early access (Dipasquale et al. 2026). These uncontrolled data require confirmation.

### 4.3 Guideline Frameworks and the Perioperative Evidence Gap

Current guidelines continue to support maximal safe resection while prioritizing functional preservation (Weller et al. 2021). The ASCO‐SNO rapid update also incorporated vorasidenib as a post‐surgical option in selected grade 2 IDH‐mutant gliomas (Blakeley et al. 2025). The perioperative safusidenib trial further explored neoadjuvant IDH inhibition between biopsy and definitive surgery (Cain et al. 2024). Despite these developments, no guideline clearly defines how surgical timing, tissue acquisition, or perioperative planning should be modified when targeted molecular therapy is incorporated into treatment pathways.

Vorasidenib does not appear to remove the rationale for cytoreduction; it may give residual disease a new role as a target for drug monitoring rather than a marker of surgical failure. Although molecular classification and targeted therapy have reshaped glioma management in this way, locoregional immunotherapy introduces a further set of operative considerations related to therapeutic delivery and surgical access. The key clinical trials and guideline frameworks informing these decisions are summarized in Table 3.

**TABLE 3:** Key clinical trials and guidelines linking molecular targeting and surgical decision‐making.

Trial/Source [Ref]	Design	Population	Key outcome	Surgical implication
INDIGO (Mellinghoff 2023) (Mellinghoff et al. 2023)	Phase III RCT	331 pts; residual/recurrent grade 2 IDH‐mutant glioma	Vorasidenib PFS 27.7 vs. 11.1 mo (HR 0.39; p < 0.0001); time to next intervention HR 0.26	Residual tumor volume remains an independent prognostic variable; GTR may alter drug‐measurable disease
CATNON methylation substudy (van den Bent 2021) (Tesileanu et al. 2021)	Retrospective substudy of Phase III	IDH‐mutant non‐codeleted anaplastic glioma	CDKN2A/B deletion, PDGFRA amp and PI3K mutations outperform histological grade	Adequate tissue for NGS/FISH is essential; incomplete specimens limit prognostication
ASCO‐SNO Rapid Update (2025) (Blakeley et al. 2025)	Guideline update	Grade 2 IDH‐mutant glioma post‐surgery	Vorasidenib recommended where RT/chemo deferred; initiation timing flexible after recovery	For GTR patients, benefit of immediate vorasidenib uncertain; no data for molecularly complete resections
EANO Guidelines (2021) (Weller et al. 2021)	Clinical guideline	IDH‐mutant WHO grade 2 astrocytoma	Maximal safe resection primary; functional preservation over EOR; watch‐and‐wait if GTR achieved	No framework for integrating IDH inhibitors into pre/intra/post‐operative planning
Perioperative safusidenib (Cain et al. 2024) (Cain et al. 2024)	Open‐label perioperative trial	Treatment‐naive low‐grade IDH‐mutant glioma	Biopsy, 28‐day IDH inhibitor, resection, continued treatment; CNS penetration assessed	Introduces a neoadjuvant IDH inhibition window; effect on tumor biology and demarcation undefined

Abbreviations: CDKN2A/B, cyclin‐dependent kinase inhibitor 2A/B; EOR, extent of resection; FISH, fluorescence in situ hybridization; GTR, gross total resection; HR, hazard ratio; IDH, isocitrate dehydrogenase; mo, months; NGS, next‐generation sequencing; PDGFRA, platelet‐derived growth factor receptor alpha; PFS, progression‐free survival; PI3K, phosphoinositide 3‐kinase; RCT, randomized controlled trial; RT, radiotherapy.

## 5 The Immunotherapy Era

### 5.1 Structural Basis of GBM Immuno‐Resistance

Glioblastoma resists systemic immunotherapy through multiple cellular and anatomical mechanisms. Tumor‐associated macrophages, microglia, myeloid‐derived suppressor cells, and regulatory T cells contribute to a highly immunosuppressive microenvironment (Liu et al. 2024; Lin et al. 2024). Glioblastoma has also been reported to reduce peripheral T‐cell activity through bone marrow sequestration mechanisms (Liu et al. 2024; Montoya et al. 2024). The blood‐brain barrier is disrupted mainly within the tumor core, whereas it remains intact at the tumor edges, where it can impede the distribution of immunotherapeutic agents (Liu et al. 2024; Arvanitis et al. 2020). Together these factors are thought to limit the effectiveness of systemic immunotherapy and support interest in locoregional treatment approaches, although the rationale differs between disease compartments, as discussed below (Lin et al. 2024; Królikowska and Tarnowski 2025).

These mechanisms are not uniform across the disease, which limits what locoregional therapy can be expected to achieve. Three compartments can be distinguished conceptually. First, the enhancing tumor bulk and the resection cavity wall, where the barrier is disrupted, and myeloid cells may account for up to half of the tumor mass (Liu et al. 2024). Their composition varies between tumors, and monocyte‐derived macrophage infiltration has been reported to be higher in IDH‐wildtype glioblastoma (Lin et al. 2024). This is the compartment removed by cytoreduction and most directly reached by catheter‐ or cavity‐based delivery. Second, the infiltrative margin, where tumor cells at the periphery are more prone to infiltrate surrounding brain tissue (Liu et al. 2024), the barrier is largely intact, and the immune environment may differ from that of the bulk. These cells are a recognized source of recurrence because infiltration limits complete resection, (Liu et al. 2024), and cells delivered to the cavity would need to migrate or diffuse to reach them. Whether they do so in effective numbers has not been established. In low‐grade glioma, tumor cells have been reported up to about 20 mm beyond MRI abnormalities (Duffau 2013), which illustrates how far infiltration can extend beyond imaging. Third, distant infiltrated brain and cerebrospinal fluid spaces. Distant parenchymal sites may more closely resemble homeostatic CNS tissue, with resident microglia rather than monocyte‐derived macrophages, but this is inferential and is not directly supported by the studies reviewed here. Cavity delivery is unlikely to reach these sites. Intraventricular delivery has been used in recurrent glioblastoma, including multifocal disease (Brown et al. 2024; Choi et al. 2024; Bagley et al. 2024), and a single‐patient report summarized in a review described regression of intracranial and spinal lesions after dual‐route delivery (Liu et al. 2024). However, we are not aware of evidence that it reaches or eliminates infiltrating tumor cells in distant brain parenchyma. Locoregional immunotherapy should therefore be viewed as addressing mainly the bulk and cavity compartments and, possibly, the margin and CSF spaces. It should not be assumed to treat the diffusely infiltrating population that contributes to recurrence. For this compartment, the surgical contribution is limited to tissue sampling and, where safe, resection of non‐enhancing tumors, which was associated with longer survival in younger IDH‐wildtype patients (Molinaro et al. 2020). Effective control would probably require systemic or multimodal approaches that remain unproven.

Table 4 summarizes major immunosuppressive mechanisms and relevant surgical considerations.

**TABLE 4:** Glioblastoma immunosuppressive microenvironment and surgical considerations.

Cell/barrier	Prevalence in GBM TME	Immunosuppressive mechanism	Surgical/perioperative consideration
TAMs/microglia	∼50% of tumor cellularity	Re‐educated by GBM toward an immunosuppressive, M2‐like phenotype with PD‐L1 expression	Debulking may reduce the TAM/microglia pool; human prospective evidence is lacking
MDSCs	4%–8% of CD45+ cells	Deplete arginine and tryptophan via arginase and IDO; drive PD‐L1‐mediated T‐cell exhaustion	Cytoreduction may reduce MDSCs (limited, mainly preclinical data)
Regulatory T cells (Tregs)	Up to 30% of TILs	GBM sequesters naïve T cells in marrow via S1P1R downregulation, depleting peripheral CD4	Debulking might reduce Treg burden; whether this improves CAR‐T function is speculative
Blood‐brain / blood‐tumor barrier	Active at non‐enhancing margins; disrupted at the CE core	Few endothelial CAMs limit immune‐cell transmigration; barrier stays intact at infiltrative margins	Locoregional delivery bypasses the barrier at the point of delivery (cavity or ventricle) but needs a surgically placed catheter or reservoir. Reach into infiltrative and distant compartments is unproven.
Dexamethasone (perioperative)	Standard perioperative agent	Expands MDSCs and depletes naïve and memory T cells; may antagonize CAR‐T engraftment	No steroid‐minimization threshold is defined and the steroid–CAR‐T efficacy relationship remains unresolved

Abbreviations: CAMs, cell adhesion molecules; CAR‐T, chimeric antigen receptor T‐cell; CE, contrast‐enhancing; GBM, glioblastoma; IDO, indoleamine 2,3‐dioxygenase; MDSCs, myeloid‐derived suppressor cells; PD‐L1, programmed death‐ligand 1; S1P1R, sphingosine‐1‐phosphate receptor 1; TAMs, tumor‐associated macrophages; TILs, tumor‐infiltrating lymphocytes; TME, tumor microenvironment.

### 5.2 CAR‐T Cell Therapy: Surgical Delivery as Pharmacological Prerequisite

Locoregional CAR‐T therapy depends on neurosurgical access for catheter or reservoir placement. IL‐13Rα2 and EGFRvIII are attractive targets because of their tumor‐associated expression patterns in glioblastoma (Liu et al. 2024; Sterner and Sterner 2024). However, systemic delivery has shown limited efficacy, probably because CAR‐T cells must cross the blood‐brain barrier and function within a suppressive tumor microenvironment (Sterner and Sterner 2024).

Recent phase I trials reported radiographic responses in some patients following intratumoral or intraventricular CAR‐T infusion (Brown et al. 2024; Geurts and Preusser 2024; Choi et al. 2024; Bagley et al. 2024; Mariniello and Migliorini 2026). In the largest study, dual intratumoral and intraventricular delivery was associated with longer median overall survival compared with single‐route delivery in a non‐randomized comparison (Brown et al. 2024). However, these findings remain hypothesis‐generating given the early‐phase, non‐randomized nature of available evidence. Surgical re‐biopsy specimens also showed evidence of antigen loss or escape after therapy in some patients (Choi et al. 2024; Bagley et al. 2024; Begley et al. 2025).

Table 5 summarizes major locoregional CAR‐T trials and their surgical implications.

**TABLE 5:** Locoregional CAR‐T trials in glioblastoma and their surgical implications.

Trial/Author [Ref]	Design	Population	Target and delivery route	Key outcome	Surgical implication
Brown et al. (2024) (Brown et al. 2024)	Phase I	Recurrent high‐grade glioma; n = 65; multiple arms	IL‐13Rα2; intratumoral, intraventricular, or dual	Dual‐route linked to longer median OS (10.2 vs. 6.1 mo single‐route); ≈50% at least stable disease	Catheter configuration (dual vs. single) may influence locoregional distribution and outcome
Choi et al. (2024), CARv3‐TEAM‐E / INCIPIENT (Choi et al. 2024)	Phase I	Recurrent GBM; n = 3	EGFRvIII via TEAM (secreted T‐cell‐engaging antibody); intraventricular (Ommaya)	Rapid radiographic regression within days; transient in 2 of 3; TIAN‐type neurotoxicity	One procedure can combine craniotomy, molecular sampling and Ommaya placement; ventricle/cavity as delivery route
Bagley et al. (2024) (Bagley et al. 2024)	Phase I (interim)	Recurrent multifocal GBM; n = 6	Bivalent EGFR + IL‐13Rα2; intrathecal / intraventricular	Early tumor size reduction; early‐onset ICANS‐type neurotoxicity	Intraventricular access required; dual‐target design intended to limit antigen escape
Kass et al. (2024) (Kass et al. 2024)	Preclinical	GBM resection‐cavity model (CLIP hydrogel)	Neural stem cell scaffold; resection‐cavity implant	>4 weeks cell persistence vs. >70% loss with free injection by day 6	Cavity geometry and implantation timing are potential operative variables

Abbreviations: CLIP, continuous liquid interface production; EGFRvIII, epidermal growth factor receptor variant III; GBM, glioblastoma; ICANS, immune effector cell‐associated neurotoxicity syndrome; IL‐13Rα2, interleukin‐13 receptor alpha 2; mo, months; OS, overall survival; TEAM, T‐cell‐engaging antibody molecule; TIAN, tumor inflammation‐associated neurotoxicity.

### 5.3 Operative Decisions and Immunotherapy Delivery: Determinants or Prerequisites?

Recent CAR‐T trials suggest that operative factors such as catheter configuration, tissue sampling, and cavity access may influence locoregional treatment delivery (Brown et al. 2024; Geurts and Preusser 2024; Choi et al. 2024). The INCIPIENT trial incorporated simultaneous craniotomy, molecular sampling, and Ommaya placement within a single procedure (Choi et al. 2024). Other studies used intraoperative FISH and postoperative cavity‐directed delivery strategies to support target confirmation and treatment administration (Bagley et al. 2024).

Preclinical scaffold‐based approaches have also suggested that cavity geometry and timing of implantation may influence cell persistence within the postoperative microenvironment (Kass et al. 2024). However, these findings remain preliminary and require prospective validation. A conceptual distinction underlies these observations and deserves to be made explicit. It is not yet clear whether operative variables such as catheter configuration, cavity geometry, and tissue handling are true determinants of immunotherapeutic efficacy or whether they are prerequisites that enable delivery without independently shaping the response. The current evidence cannot separate the two, because in the available early‐phase trials surgical access and treatment delivery are confounded by design: an apparent difference between delivery routes may reflect the route itself, the tumors that happened to be amenable to it or unmeasured biological factors. Until comparative studies are designed to isolate the operative variable, the neurosurgical contribution to locoregional immunotherapy is best regarded as a plausible but unproven determinant of efficacy rather than an established one. These considerations apply mainly to the bulk, cavity, and CSF compartments. None of the cited trials was designed to assess activity against distant infiltrating disease. Figure 2 summarizes the neurosurgeon's operative role in locoregional immunotherapy as a decision algorithm.

**FIGURE 2:**
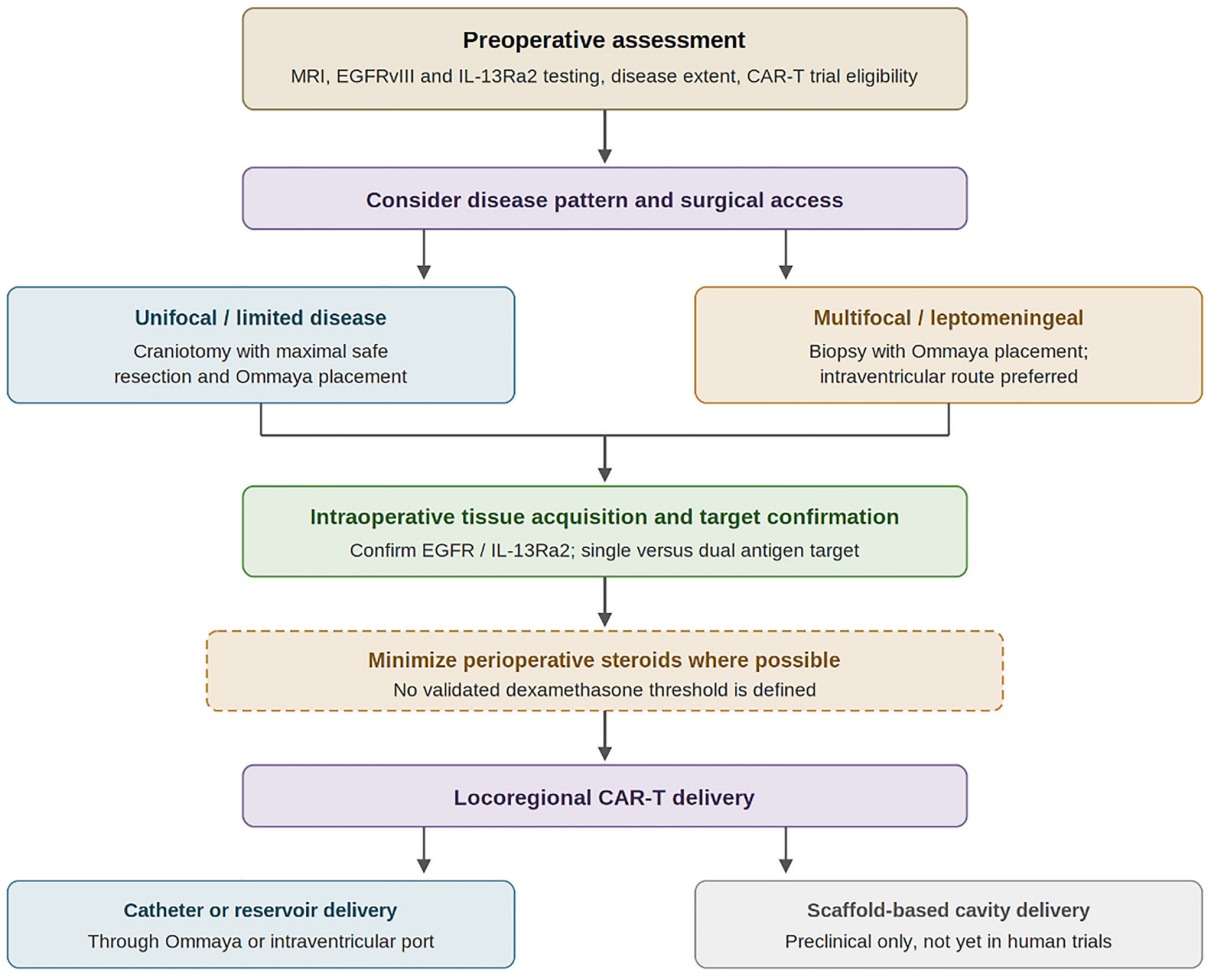
Potential Surgical Pathways Supporting Locoregional Immunotherapy Delivery: A Conceptual Framework; Applies to bulk, cavity and CSF compartments; distant infiltrating disease is not addressed. Alt text: Flowchart of surgical pathways supporting locoregional immunotherapy delivery in glioblastoma. Preoperative assessment (MRI, EGFRvIII and IL‐13Rα2 testing, disease extent, CAR‐T eligibility) leads to a disease‐pattern decision: unifocal or limited disease (craniotomy with maximal safe resection and Ommaya placement) vs. multifocal or leptomeningeal disease (biopsy with Ommaya placement, intraventricular route preferred). Both converge into intraoperative tissue acquisition and target confirmation, confirming EGFR and IL‐13Rα2 status and single vs. dual antigen targeting. A dashed step highlights minimizing perioperative steroids, noting no validated dexamethasone threshold exists. The pathway reaches locoregional CAR‐T delivery, branching into catheter or reservoir delivery (via Ommaya or intraventricular port) vs. scaffold‐based cavity delivery, marked as preclinical only and not yet in human trials. Labeled a conceptual, non‐validated synthesis.

Whether the resection cavity should be planned as a delivery site remains a hypothesis rather than established practice, but it is one with enough early‐phase support to warrant testing. Either way, the expanding surgical responsibilities introduced by molecular characterization and locoregional therapeutic delivery have increased dependence on intraoperative technologies capable of simultaneously maximizing resection, preserving function, and facilitating precise tissue acquisition.

## 6 Current Controversies

The developments discussed above have not produced full agreement. Instead, they have created several open questions. Five of these are important because each one depends on a surgical decision, not only on an oncological one (Table 6).

**TABLE 6:** Current controversies in glioma surgical decision‐making.

Controversy	Position taken	Rationale and evidence
1. Does vorasidenib reduce the need for aggressive resection?	No — but it changes the calculus for borderline cases	Where gross total resection is achievable without functional compromise, residual tumor volume retains independent prognostic significance. Where it is not safely achievable, residual disease doubles as a measurable target for vorasidenib monitoring. The drug does not appear to replace resection; it may provide a rationale for accepting planned subtotal resection.
2. Is supratotal resection still justified in all IDH‐mutant gliomas?	Not universally	Prospective series support supratotal resection in non‐eloquent low‐grade glioma. But with vorasidenib now offering disease control, the marginal oncological benefit of resecting beyond functional boundaries is no longer self‐evident and has not been tested in the post‐INDIGO setting.
3. Can molecular therapy compensate for residual disease?	Unknown — and INDIGO cannot answer it	INDIGO enrolled patients with residual or recurrent disease. Its progression‐free survival benefit does not establish equivalence between gross total resection followed by vorasidenib and deliberate subtotal resection followed by vorasidenib. This is the central unanswered question in IDH‐mutant glioma surgery.
4. Should locoregional immunotherapy delivery become a surgical endpoint?	Premature to mandate, appropriate to plan for	Early‐phase data suggest catheter configuration and may influence delivery (cavity‐geometry data are preclinical only) but cannot separate operative variables from tumor biology, because surgical access and delivery are confounded by design. Surgeons should plan for immunotherapy access without treating delivery optimization as an established objective. Even if delivery is optimized, it addresses mainly the bulk, cavity and CSF compartments, and infiltrating cells at distant sites are unlikely to be reached.
5. Are imaging‐based resection limits becoming outdated?	Not entirely; the field is evolving	The EANS‐EANO 2025 guidelines and the RANO resect classification reflect a shift toward biologically and functionally informed resection endpoints, which increasingly complement fixed radiographic thresholds. Imaging itself is also advancing (non‐enhancing tumor, intraoperative imaging, fluorescence guidance, and radiomics), and advanced modalities such as MR spectroscopy, metabolic imaging and 7T MRI may refine resection limits further but were not assessed in this review. The transition is incomplete, and its direction is not yet settled.

Abbreviations: EANO, European Association of Neuro‐Oncology; EANS, European Association of Neurosurgical Societies; GTR, gross total resection; IDH, isocitrate dehydrogenase; RANO, Response Assessment in Neuro‐Oncology.

### 6.1 Does Vorasidenib Reduce the Need for Aggressive Resection?

Not directly, but it changes the decision in difficult cases. When gross total resection is possible without harming function, residual tumor volume still has prognostic value, so maximal resection may remain the preferable option. When gross total resection is not safe, the remaining tumor can be used as a target for vorasidenib monitoring. In this way, the drug does not appear to replace surgery; it may instead provide a reason to accept planned subtotal resection when needed.

### 6.2 Is Supratotal Resection Still Useful in all IDH‐Mutant Gliomas?

Not in every case. Some prospective studies have supported supratotal resection in low‐grade glioma in non‐eloquent areas. But now that vorasidenib can control the disease, the extra benefit of removing tissue beyond the functional limit is no longer clear. This question has not been studied after the INDIGO trial.

### 6.3 Can Molecular Therapy Make Up for Residual Disease?

This is not known, and the INDIGO trial cannot answer it. The trial included patients who already had residual or recurrent disease. Therefore, its benefit does not prove that gross total resection followed by vorasidenib and planned subtotal resection followed by vorasidenib give the same result. This remains the most important open question in IDH‐mutant glioma surgery.

### 6.4 Should Locoregional Immunotherapy Delivery Become a Surgical Goal?

It is too early to require this, but it is reasonable to plan for it. The question must be framed with attention to glioblastoma biology, because the resection cavity addresses only part of the disease. Locoregional delivery acts mainly on the enhancing bulk and cavity wall, where the barrier is disrupted, (Liu et al. 2024; Arvanitis et al. 2020) and, through intraventricular routes, on the CSF spaces (Brown et al. 2024; Choi et al. 2024; Bagley et al. 2024). It is not expected to reach the diffusely infiltrating cells in distant parenchyma, which are a recognized source of recurrence. (Liu et al. 2024) Even in low‐grade glioma, tumor cells have been reported beyond MRI abnormalities, (Duffau 2013) which illustrates how far infiltration can extend beyond what imaging shows. Early studies suggest that catheter configuration and cavity access may affect delivery, (Brown et al. 2024; Geurts and Preusser 2024; Choi et al. 2024) but they cannot separate surgical factors from tumor biology because access and delivery are linked by trial design. Delivery should therefore be regarded as one component of a multimodal strategy, not as a surgical goal that can compensate for residual infiltrative disease. Surgeons should prepare for immunotherapy access, but the contributions on firmer ground are different: maximal safe resection, including non‐enhancing tumor where safe in selected IDH‐wildtype patients; (Molinaro et al. 2020) adequate tissue acquisition to confirm target antigens, since antigen loss or escape was observed at re‐biopsy; (Choi et al. 2024; Bagley et al. 2024; Begley et al. 2025) and selection of the delivery route according to disease pattern, with intraventricular delivery used in multifocal disease (Brown et al. 2024; Choi et al. 2024; Bagley et al. 2024). Control of distant infiltrating disease would probably require systemic or multimodal approaches that remain unproven.

### 6.5 Are Imaging‐Based Resection Limits Becoming Outdated?

Not entirely, and the direction is still evolving. The EANS‐EANO 2025 guidelines and the RANO resect classification show a move toward resection goals informed by biology and function, in addition to fixed imaging cut‐offs (Goldbrunner et al. 2025; Wen et al. 2025). Molecular testing, preservation of function, and treatment delivery increasingly complement the older percentage thresholds. This should not be read as imaging losing its role. Volumetric imaging remains the basis for defining and comparing extent of resection, and its limits are being addressed from several directions: inclusion of non‐enhancing tumors in resection targets (Molinaro et al. 2020); recognition that MRI underestimates infiltration (Duffau 2013); intraoperative imaging and 5‐aminolevulinic acid fluorescence that improve detection of residual tumor and complete resection (Stummer et al. 2006; Roder et al. 2023; Fountain et al. 2021); and radiomic and artificial intelligence approaches that remain investigational (Sanker et al. 2026; Patel et al. 2026). Advanced modalities such as MR spectroscopy, metabolic imaging, and ultra‐high‐field (7T) MRI may sharpen the definition of tumor extent further. Their role in defining resection limits was not evaluated in this review and warrants prospective study. The field is dynamic, and improved diagnostics may refine and redefine imaging‐based resection limits, not simply replace them.

## 7 Intraoperative Technologies and Functional Preservation

Modern glioma surgery increasingly depends on intraoperative technologies that support maximal safe resection while preserving neurological function. These approaches also improve tissue acquisition and operative precision in the molecular era.

Fluorescence‐guided surgery with 5‐aminolevulinic acid was associated with higher complete resection rates and improved 6‐month progression‐free survival in a phase III trial compared with white light surgery in glioblastoma (Stummer et al. 2006). In low‐grade glioma its diagnostic value appears greater than its resective utility because visible fluorescence is less common (Picart et al. 2024).

Intraoperative MRI has been reported to improve detection of residual tumor and compensate for brain shift, although a prospective trial found it not superior to 5‐ALA guidance in glioblastoma (Roder et al. 2023; Fountain et al. 2021). Intraoperative ultrasound provides a more accessible real‐time alternative in centers without intraoperative MRI capability (Picart et al. 2024). Current evidence suggests that both imaging approaches improve operative guidance, although confirmed survival benefit remains uncertain (Roder et al. 2023; Fountain et al. 2021).

Awake craniotomy with direct electrostimulation remains central for surgery involving eloquent cortex (Hervey‐Jumper and Berger 2016; De Witt Hamer et al. 2012). Meta‐analytic evidence suggests improved extent of resection with lower permanent neurological deficit rates when mapping techniques are used (De Witt Hamer et al. 2012; Baig Mirza et al. 2025). In the cited series, MEP amplitude changes were irreversible in approximately 40% of cases, requiring real‐time team interpretation as an active resection determinant (Guzzi et al. 2024).

Functional preservation remains important because postoperative neurological deficits may reduce tolerance to adjuvant therapy and negatively affect quality of life (Pöhlmann et al. 2024; Hervey‐Jumper and Berger 2016; De Zwart and Ruis 2024). Adequate tissue acquisition and preservation of neurological function are therefore both important components of treatment planning.

### 7.1 Artificial Intelligence and Surgical Planning

Artificial intelligence applications in glioma surgery continue to expand. Current research focuses mainly on radiogenomic prediction, operative planning, and intraoperative imaging analysis (Sanker et al. 2026; Patel et al. 2026).

Radiomic models have shown potential for predicting IDH mutation, MGMT methylation, and 1p19q codeletion using preoperative imaging (Sanker et al. 2026). These methods may support biopsy targeting and surgical planning, although molecular pathology remains necessary for diagnosis and treatment selection.

Artificial intelligence has also been explored for intraoperative navigation and tissue characterization (Sanker et al. 2026; Patel et al. 2026). However, most systems remain investigational and lack prospective multicenter validation. Current evidence supports their role as adjunctive decision support tools rather than independent determinants of operative strategy.

### 7.2 Functional Outcomes and Quality of Life

Functional outcomes remain central in glioma surgery because postoperative neurological deficits directly affect independence, cognition, and quality of life (Pöhlmann et al. 2024; Hervey‐Jumper and Berger 2016). Preservation of language, motor, and cognitive function is therefore an essential operative objective in its own right.

This matters most in low‐grade glioma, where survival can extend for many years and where long‐term cognitive and occupational function, rather than short‐term resection metrics, often determines whether a patient returns to work and independent living. Here the aim of surgery is not simply to maximize resection but to protect the neurological reserve on which a long survivorship depends.

The balance between aggressive cytoreduction and neurological preservation therefore remains individualized, depending on tumor location, molecular subtype, patient age, and anticipated adjuvant therapy.

### 7.3 Technological Limitations and Access Disparities

Despite advances in intraoperative imaging, major differences remain in access to neurosurgical technologies across healthcare systems (Roder et al. 2023; Fountain et al. 2021). Intraoperative MRI, fluorescence‐guided surgery, and advanced neurophysiological monitoring are often limited to specialized centers.

Cost, infrastructure requirements, and operative time may restrict implementation in resource‐limited settings. Variability in institutional expertise also affects outcomes and limits standardization across studies.

As molecular and immunotherapeutic strategies continue to evolve, disparities in access to advanced surgical technologies may contribute to unequal treatment opportunities.

## 8 Discussion

Glioma surgery has evolved beyond a purely anatomical model focused only on cytoreduction (Sanai and Berger 2018). Molecular classification‐targeted therapy and locoregional immunotherapy have introduced additional considerations related to tissue acquisition, treatment sequencing, and therapeutic delivery.

When we look at all this evidence together, the goal of surgery has not changed in its main idea, but it has changed in how it is used in practice. Retrospective studies and meta‐analyses consistently report an association between greater extent of resection and longer survival in both high‐grade and low‐grade glioma, although confounding by indication cannot be excluded (Lacroix et al. 2001; Sanai et al. 2011; Brown et al. 2016; Jagtiani et al. 2024; Xia et al. 2018). However, many foundational extent of resection studies were conducted before routine molecular classification and did not account for biomarkers such as IDH mutation, MGMT methylation, or other molecular subtypes (Hervey‐Jumper and Berger 2016). Contemporary operative planning therefore increasingly depends on integration of both radiographic and molecular information.

The introduction of IDH inhibitors has also changed management considerations in selected IDH‐mutant gliomas (Mellinghoff et al. 2023; Van Den Bent et al. 2024; Blakeley et al. 2025). Residual tumor volume remains prognostically important while targeted therapies now provide additional treatment options for selected patients. Questions regarding operative timing, postoperative treatment sequencing, and optimal residual disease burden remain incompletely resolved. The most important open question is whether planned subtotal resection with vorasidenib can give the same result as gross total resection. This has not been tested directly yet.

Locoregional CAR T approaches have further increased the importance of operative access, catheter placement and tissue acquisition (Brown et al. 2024; Geurts and Preusser 2024; Choi et al. 2024; Bagley et al. 2024). Early‐phase studies suggest that surgical variables may influence therapeutic delivery and monitoring, although prospective validation remains limited. It is too early to say that the delivery route alone decides how well immunotherapy works. In the current trials, the surgical access and the tumor type are linked, so their effects cannot be separated.

Current evidence supports a broader surgical planning approach in which molecular subtype, functional preservation, tissue acquisition, and therapeutic delivery are considered together during operative decision‐making.

The oncofunctional‐immunotherapeutic framework proposed here operates across three planning dimensions. First, molecular subtype functions as a pre‐operative planning variable: IDH mutation status, MGMT methylation, and CDKN2A/B deletion may inform resection targets, tissue acquisition priorities, and adjuvant sequencing. Second, the resection cavity is treated as a therapeutic instrument for the bulk and cavity compartments: catheter placement, cavity geometry, and steroid minimization are operative decisions that may influence locoregional immunotherapy delivery. Third, functional preservation is positioned as a prerequisite for downstream therapy rather than a secondary objective: neurological deficits that limit adjuvant eligibility may represent a failure of surgical planning rather than merely a complication. The clearest objection to this framework is that it formalizes relationships that are still largely unproven, most of all the cavity‐as‐instrument dimension, which currently rests on early‐phase and preclinical data. We therefore present it not as a validated protocol but as a hypothesis‐generating structure, intended to make these emerging surgical decisions explicit and testable rather than to prescribe them.

### 8.1 Clinical Implications

Several practical implications emerge from current evidence.

First, adequate tissue acquisition is now essential because molecular biomarkers directly influence diagnosis, prognosis, and treatment eligibility (Louis et al. 2021; Juratli et al. 2023; Guo et al. 2024). Second, operative planning increasingly depends on balancing cytoreduction with preservation of neurological function and future treatment options. Third, locoregional immunotherapy approaches may require coordinated planning between neurosurgeons, neuro‐oncologists, and molecular pathology teams.

These developments support a multidisciplinary treatment approach in which surgery functions as part of an integrated therapeutic strategy.

### 8.2 Future Directions

Future glioma surgery will likely become increasingly integrated with molecular diagnostics, targeted therapies, and locoregional immunotherapy approaches. Prospective studies are needed to determine how molecular subtype should influence extent of resection goals and perioperative treatment strategies.

Further research is also required to evaluate neoadjuvant targeted therapy, cavity‐directed therapeutic delivery, scaffold‐based cellular therapies, artificial intelligence‐assisted operative planning, and standardized functional outcome assessment.

As locoregional immunotherapy strategies continue to evolve, operative variables such as catheter placement, delivery route, and postoperative cavity architecture may become increasingly important areas of investigation. Future locoregional trials should report outcomes by compartment, distinguishing local cavity recurrence from distant parenchymal and leptomeningeal progression, so that the reach of these therapies can be defined. The SNO‐EANO consensus also proposed the RANO resect classification to standardize extent of resection terminology across future trials (Wen et al. 2025). Studies should also evaluate whether advanced imaging, such as MR spectroscopy, metabolic imaging, and 7T MRI, can refine imaging‐based resection limits.

### 8.3 Future Clinical Trial Priorities

The questions raised in this review can be turned into clear research priorities. We list five that we consider most important (Table 7).

**TABLE 7:** Future clinical trial priorities.

Priority	Rationale and proposed design
1. Molecularly stratified extent‐of‐resection trials	Prospective studies comparing oncological outcomes across defined resection thresholds within IDH‐mutant and IDH‐wildtype subgroups separately rather than pooling them. The RANO resect classification offers a standardized framework for endpoints.
2. Post‐vorasidenib residual volume studies	Trials examining whether the prognostic impact of residual disease volume is modified by vorasidenib. Whether planned subtotal resection followed by vorasidenib is equivalent to gross total resection requires direct, prospective comparison.
3. Neoadjuvant IDH inhibitor window studies	Building on the perioperative safusidenib model to determine whether pre‐surgical IDH inhibition alters tumor demarcation, reduces infiltrative margin, or improves subsequent resection completeness.
4. Surgical optimization of CAR‐T delivery	Prospective studies treating operative variables — catheter configuration, cavity geometry, perioperative steroid tapering — as primary independent variables rather than confounders. Current evidence confounds delivery route with tumor biology.
5. Biological extent‐of‐resection metrics	Development and validation of resection endpoints beyond radiographic volumetrics: circulating tumor DNA clearance, cerebrospinal fluid proteomics, or intraoperative molecular sampling as surrogates for biologically complete resection.

Abbreviations: CAR‐T, chimeric antigen receptor T‐cell; IDH, isocitrate dehydrogenase; RANO, Response Assessment in Neuro‐Oncology.

First, future trials should study the extent of resection separately in IDH‐mutant and IDH‐wild‐type tumors, instead of mixing them together. The RANO resect classification can be used to keep the resection categories the same across studies.

Second, studies are needed to see whether the effect of residual tumor volume changes when patients receive vorasidenib. The INDIGO trial only included patients who already had residual disease, so we still do not know if planned subtotal resection followed by vorasidenib yields the same result as gross total resection.

Third, neoadjuvant trials should test whether giving an IDH inhibitor before surgery changes the tumor border or makes the later resection more complete. The safusidenib study is an early example of this approach.

Fourth, trials on CAR‐T delivery should treat surgical factors such as catheter position, cavity shape, and steroid use as the main variables to study, not as side factors. In current studies these factors are mixed with tumor biology, so their real effect is not clear.

Fifth, new ways to measure resection should be developed and tested. These may include circulating tumor DNA, cerebrospinal fluid markers, or molecular testing during surgery, which could show whether the resection is complete in a biological sense and not only on imaging.

### 8.4 Limitations

This review has several limitations. Much of the evidence regarding the extent of resection and locoregional immunotherapy remains retrospective or early‐phase in nature. Considerable heterogeneity also exists across operative techniques, molecular classification systems, and patient populations. The proposed framework addresses mainly the tumor bulk and resection cavity and offers no solution for diffusely infiltrating disease distant from the cavity, which is thought to contribute to recurrence. Advanced imaging modalities, including MR spectroscopy, metabolic imaging and 7T MRI, were not systematically assessed.

The proposed oncofunctional immunotherapeutic framework has not been prospectively validated and should therefore be interpreted as a conceptual synthesis of currently available evidence.

Narrative reviews are also inherently subject to selection bias despite structured literature searching. The review is limited to English‐language publications; gray literature was excluded, and the proposed framework has not undergone formal expert consensus validation. Study selection and evidence appraisal were performed without independent duplication, and no formal risk‐of‐bias tool was applied. Evidence was instead graded using a pragmatic classification, so some degree of selection and interpretive bias is likely. The literature search was completed in May 2026, and later publications are not captured.

## 9 Conclusion

Glioma surgery remains centered on maximal safe resection, but modern operative planning increasingly requires integration of molecular classification, targeted therapy and locoregional immunotherapy considerations.

The molecular era has increased the importance of tissue acquisition, perioperative planning, and functional preservation. Emerging locoregional immunotherapy approaches have also increased the relevance of surgical access and cavity‐directed therapeutic delivery. The resection cavity can be viewed as a therapeutic instrument for the bulk and cavity compartments, with catheter placement, cavity geometry, and steroid minimization representing operative considerations that may influence locoregional immunotherapy delivery.

Current guidelines do not fully integrate these developments into a unified surgical decision‐making framework. This review proposes a conceptual framework that combines molecular profiling, functional preservation, and therapeutic delivery considerations within contemporary glioma surgery.

## Author Contributions

Conceptualization of ideas: **Naeem Ul Haq**, **Sardar Umer Irshad**, **Irfan Ullah**, **Shah Mir**, **Jahan Mubariz**. Manuscript preparation: **Sardar Umer Irshad**, **Naeem Ul Haq**, **Sardar Remsha Irshad**, **Shah Mir**, **Ali Mumtaz**. Writing of initial draft: **Sardar Umer Irshad**, **Muhammad Shaheer Khan**, **Irfan Ullah**, **Malik Ahsan Jamal**. Review and editing: **Sardar Umer Irshad**, **Muhammad Shaheer Khan**, **Sardar Remsha Irshad**, **Malik Ahsan Jamal**. Validation: **Jahan Mubariz**, **Ali Mumtaz**, **Sardar Umer Irshad**, **Malik Ahsan Jamal**. Final Draft: All authors approved the final manuscript.

## AI Disclosure

AI‐based tools were used only to generate illustrative artwork for the figures and the graphical abstract. The content of these visuals was specified by the authors from the text of this manuscript. No patient data, unpublished data or research results were entered into any AI tool, and AI was not used to generate, alter or analyze data. All AI‐generated visuals were reviewed by the authors and checked against the manuscript for accuracy. The authors take full responsibility for the integrity of the content. Reporting follows the TITAN guideline (Agha et al. 2025).

## Funding

The authors have nothing to report.

## Conflicts of Interest

The authors declare no conflicts of interest.

## Data Availability

Data sharing is not applicable to this article as no datasets were generated or analyzed during the current study.
